# The Tumor Suppressor Roles of miR-433 and miR-127 in Gastric Cancer

**DOI:** 10.3390/ijms140714171

**Published:** 2013-07-08

**Authors:** Li-Hua Guo, Hui Li, Fang Wang, Jia Yu, Jin-Sheng He

**Affiliations:** 1 School of Computer and Information Technology, College of Life Sciences and Bioengineering, Beijing Jiaotong University, 3 Shangyuan Residence, Haidian District, Beijing 100044, China; E-Mail: lihuaguo@bjtu.edu.cn; 2 Department of Critical Care Medicine (CCM), Peking University People’s Hospital, Beijing 100044, China; E-Mail: haitilee@163.com; 3 Institute of Basic Medical Sciences, Peking Union Medical College & Chinese Academy of Medical Sciences, Beijing 100005, China; E-Mail: wo_wfang@hotmail.com

**Keywords:** gastric cancer, miR-433, miR-127, KRAS, MAPK4

## Abstract

The discovery of microRNAs (miRNAs) provides a new and powerful tool for studying the mechanism, diagnosis and treatment of human cancers. Currently, the methylation epigenetic silencing of miRNAs with tumor suppressor features by CpG island hypermethylation is emerging as a common hallmark of different tumors. Here we showed that miR-433 and miR-127 were significantly down-regulated in gastric cancer (GC) tissues compared with the adjacent normal regions in 86 paired samples. Moreover, the lower level of miR-433 and miR-127 was associated with pM or pTNM stage in clinical gastric cancer patients. The restored expression of miR-433 and miR-127 in GC cells upon 5-Aza-CdR and TSA treatment suggested the loss of miR-433 and miR-127 was at least partly regulated by epigenetic modification in GC. Furthermore, the ectopic expression of miR-433 and miR-127 in gastric cancer cell lines HGC-27 inhibits cell proliferation, cell cycle progression, cell migration and invasion by directly interacting with the mRNA encoding oncogenic factors KRAS and MAPK4 respectively. Taken together, our results showed that miR-433 and miR-127 might act as tumor suppressors in GC, and it may provide novel diagnostic and therapeutic options for human GC clinical operation in the near future.

## 1. Introduction

Gastric cancer (GC) is one of the most common types of cancer worldwide, with particularly high incidences and mortality rates in eastern Asia [1]. Over the past decades, great effort has been exerted to elucidate the underlying mechanisms and to discover novel diagnostic biomarkers and therapeutic targets for GC patients. However, the molecular mechanism underlining GC tumorigenesis rate remains largely unknown.

Recently, the classical categories of oncogenes and tumor suppressor genes have been expanded to include a new family of RNAs known as microRNAs (miRNAs), which may regulate a vast number of protein-coding genes, including tumor-related genes [2–5]. Increasing evidences have highlighted the changes in miRNA expression that could be associated with cancer initiation and progression, as well as specific pathologic features [6,7]. Moreover, better knowledge of changes in miRNA gene expression during carcinogenesis may lead to improvements in the disease characteristics and therefore be used to inform GC patient management.

MiR-433 and miR-127 are derived from an overlapping gene locus highly conserved among different mammalian species. These two miRNA genes are expressed from distinct primary miRNAs (pri-miR-433 and pri-miR-127) encoded by the miR-433-127 locus [8]. They co-localized on chromosome 14q32, a region that is often involved in several types of translocations in hematological malignancies and deleted by loss of heterozygosity (LOH) in solid tumors [8]. A recent report has indicated the decreased expression of miR-127 in hepatocellular carcinoma [9]. Luo and Ueda had reported the down-regulation of miR-433 in gastric carcinoma, respectively [10,11]; another study showed that miR-433 was up-regulated in CR-ABL-negative myeloproliferative neoplasms [12]. However, the potential roles of miR-433 and miR-127 in GC have largely undefined. Here, we analyzed the expression level of miR-433 and miR-127 in 86 paired GC tissues and adjacent normal tissues, and demonstrated that miR-433 and miR-127 were both down-regulated in GC tissues compared with normal control, as well as in GC cell lines HGC-27, MGC-803, MKN-45 and SGC-7901. The silenced expression of miR-433 and miR-127 in HGC-27 and MGC-803 cells was restored by the DNA methyltransferase inhibitor 5-aza-2′-deoxycytidine (5-Aza-CdR) and histone-deacetylase inhibitor trichostatin A (TSA) treatment, suggesting the epigenetic regulation of this miRNA cluster in GC cells. Furthermore, the restored miR-433 and miR-127 expression upon miRNA mimics transfection inhibited cell proliferation, cell cycle and migration in HGC-27 cell. The tumor suppressor roles of miR-433 and miR-127 in GC cells were mediated by inhibition of KRAS and MAPK4, respectively. These results suggested that the miR-433~miR-127 cluster function as tumor suppressor miRNAs in GC and provided potential therapy strategy for GC patients by targeting miRNA expression.

## 2. Results and Discussion

### 2.1. MiR-433 and miR-127 Were Down-Regulated in GC Patients and GC Cell Lines

**Figure 1:**
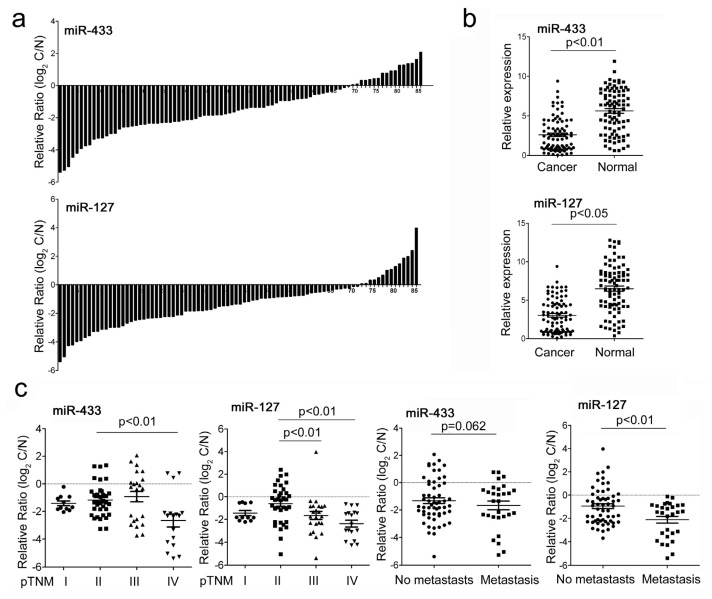

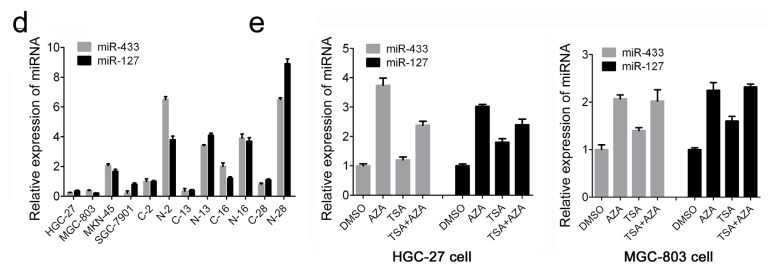
The expression of miR-433 and miR-127 in gastric cancer (GC) tissues and cell lines. (**a**) MiR-433 and miR-127 were detected in 86 pairs of GC tissues and its adjacent normal controls by quantitative RT-PCR. Data are presented as log2 of fold change of GC tissues relative to adjacent normal regions; (**b**) Relative miR-433 and miR-127 expression levels in GC tissues and adjacent normal regions; (**c**) The Statistical analysis of the association between miRNA level and pTNM stage (I, II, III and IV) and pM stage (No metastasis and Metastasis); (**d**) The relative level of miR-433 and miR-127 in GC cell lines (HGC-27,MGC-803, MKN-45 and SGC-7901) relative to four paired samples; (**e**) The expression of miR-433 and miR-127 in HGC-27 and MGC-803 cells after treatment with AZA,TSA or AZA and TSA.

The knowledge that miRNAs coming from a same cluster can reinforce their action on common cellular pathways lead us to investigate the association of miR-433~miR-127 cluster with gastric carcinogenesis. To evaluate the expression of miR-433 and miR-127, quantitative RT-PCR analysis was performed in 86 pairs of clinic GC tissue and matched adjacent normal tissue samples. Expression levels of miR-433 and miR-127 were much lower in tumor than in non-tumor tissues (Figure 1a,b). To further study the relationship between miR-433/miR-127 expression and clinicopathological factors, the level of miR-433/miR-127 in GC tissues (including fully clinical information) were statistically analyzed (non-parametric test). The lower level expression of miR-127 was associated with pM stage (*p* = 0.009, metastasis *vs.* no metastssis), and lower level of miR-127 was associated with pTNM stage (*p* = 0.009, stage II *vs.* III, *p* = 0.008, stage II *vs.* IV) in GC patients (Figure 1c). The examination of miR-433 and miR-127 expression in four GC cell lines (HGC-27, MGC-803, MKN-45 and SGC-7901) also indicated the down-regulation when compared to non-tumor tissues (Figure 1d). These data suggest that alterations of miR-433 and miR-127 expression might be involved in gastric cancer progression. A previous study has indicated the association of miR-127 with methylation in GC [13]. In an attempt to reveal the underlining mechanisms regulating both miR-433 and miR-127 expression in GC cells, the DNA methyltransferase inhibitor 5-aza-2′-deoxycytidine (5-Aza-CdR) and histone-deacetylase inhibitor trichostatin A (TSA) were used to detect whether the miR-433~miR-127 locus was epigenetically modified in GC. Quantitative RT-PCR results showed that the expression of miR-433 and miR-127 was consistently increased in two situations (Figure 1e): for the 5-Aza-CdR treatment, the expression of miRNA was up-regulated in HGC-27 (5-Aza-CdR 0.6μmol/L; 3.74-fold for miR-433 and 3.02-fold for miR-127) and MGC-803 (5-Aza-CdR 1.5 μmol/L; 2.07-fold for miR-433 and 2.25-fold for miR-127) compared with DMSO control group; for the 5-Aza-CdR and TSA combination treatment, the expression of miR-433 is much higher in HGC-27 (5-Aza-CdR 0.6 μmol/L; 1.98-fold for miR-433 and 1.33-fold for miR-127) and MGC-803 (5-Aza-CdR 1.5 μmol/L; 1.43-fold for miR-433 and 1.45-fold for miR-127) compared with TSA control group. However, the alone treatment of TSA and the combinational treatment of 5-Aza-CdR and TSA didn’t affect the level of miR-433 and miR-127 in both cell lines. These results indicated that the dysregulation of miR-433 and miR-127 in GC may be restricted to the hypermethylation of CpG-rich regions in their genomic locus.

### 2.2. Increased miR-433 and miR-127 Inhibits Cell Proliferation and Cell Cycle Progression of GC Cells

**Figure 2:**
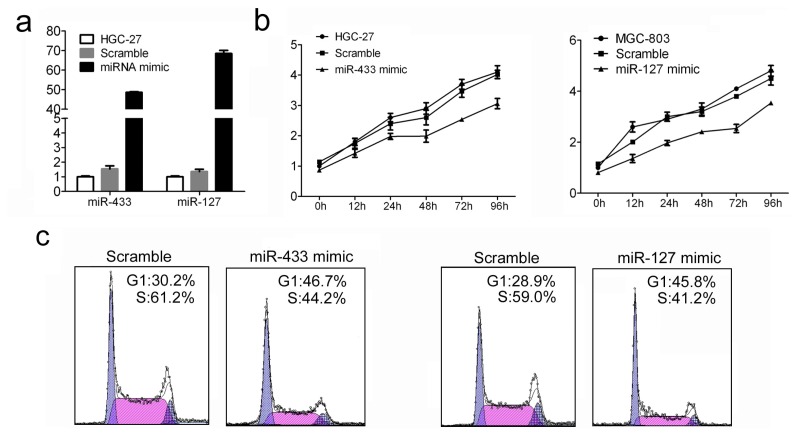
The influence of miR-433 and miR-127 on GC cell proliferation and cell cycle progression. (**a**) MiR-433 and miR-127 level were detected in HGC-27 cells after treatment with miRNA mimic (30 nM) or Scramble (30 nM) by quantitative RT-PCR; (**b**) Cell proliferation assay of HGC-27 cells after treatment with miRNA mimic or Scramble by using CCK-8; (**c**) Cell cycle analysis of HGC-27 cells after treatment with miRNA mimic or Scramble by Propidium Iodide (PI) staining.

The remarkable reduction of miR-433 and miR-127 expression in GC patients promotes us to explore the possible biological significance of these miRNAs in tumorigenesis. To study the relevance of miR-433/miR-127 level and GC cell growth, miRNA mimics or scramble were used to transfect into HGC-27 cells. The intracellular level of miR-433 and miR-127 was about 40-fold and 60-fold higher in HGC-27 cells treated with miRNA mimics than scramble, respectively (Figure 2a). The proliferation of transfected HGC-27 cells was measured by using CCK-8 assay. Enforced expression of miR-433 and miR-127 led to significant decrease in cell proliferation in HGC-27 cells (Figure 2b). Accordingly, the percentage of S phase cells was also reduced by ~17% and ~16% in HGC-27 cells treated with miR-433 mimic and miR-127 mimic respectively (Figure 2c). Taken together, these results indicated that miR-433 and miR-127 can efficiently inhibited tumor cell proliferation and cell cycle *in vitro*.

### 2.3. Over-Expression of miR-433 and miR-127 in GC Cells Inhibits Cell Migration and Invasion

**Figure 3:**
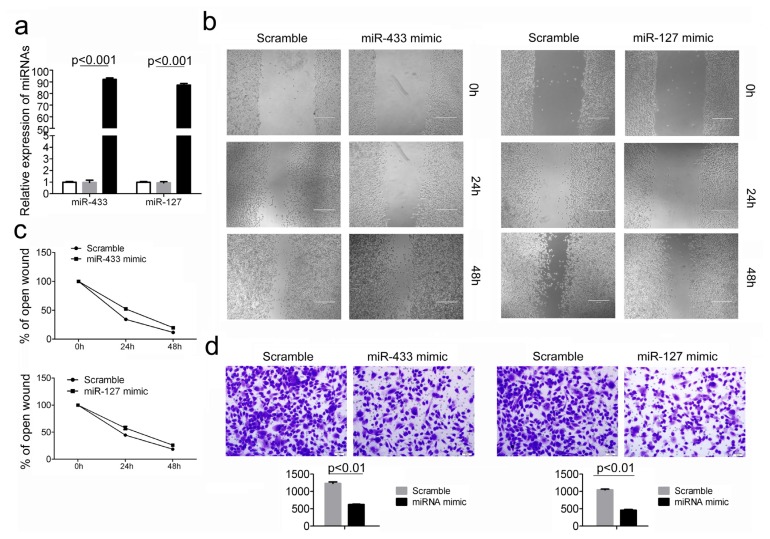
The influence of miR-433 and miR-127 on GC cell migration and invasion. (**a**) MiR-433 and miR-127 level were detected in HGC-27 cells after treatment with miRNA mimic or Scramble by uantitative RT-PCR; (**b**,**c**) Wound healing assays of HGC-27 cells after treatment with miRNA mimic or Scramble, The relative ratio of wound closure per field was shown in (c); (**d**) Transwell analysis of HGC-27 cells after treatment with miRNA mimic or Scramble, The relative ratio of invasive cells per field was shown below.

Cell migration and invasion promote tumor metastasis, which is the major cause of GC morbidity and death [14]. Given that miR-433 and miR-127 display close association with metastasis and malignant degree of tumor, we proposed that these two miRNAs may play an extremely important role in GC migration and invasion. To test this hypothesis, cell migration and invasion assays were performed in HGC-27 cells transfected with either miR-433 mimic or miR-127 mimic, and scramble controls. Quantitative RT-PCR analysis showed that the miRNA mimic treatment led to more than 100-fold increase of miR-433 and miR-127 level in HGC-27 cells (Figure 3a). Therefore, the introduction of miR-433 and miR-127 into HGC-27 cells resulted in a significant reduction of cell migration during the closing of an artificial wound created over a confluent monolayer (Figure 3b,c). Moreover, these cells were maintained in serum-free medium during the course of wound healing to ensure that any augmented migratory behavior could not be affected by altered cell proliferation. In addition, restored expression of miR-433 and miR-127 dramatically inhibited the normally strong invasive capacity of HGC-27 cells as indicated in the transwell invasion assay (Figure 3d). These results were consistent with the above findings that the expression of miR-433 and miR-127 were much lower in GC patients with more malignant degree.

### 2.4. Restored miR-433 Expression Dampens KRAS Pathway in GC Cells

**Figure 4:**
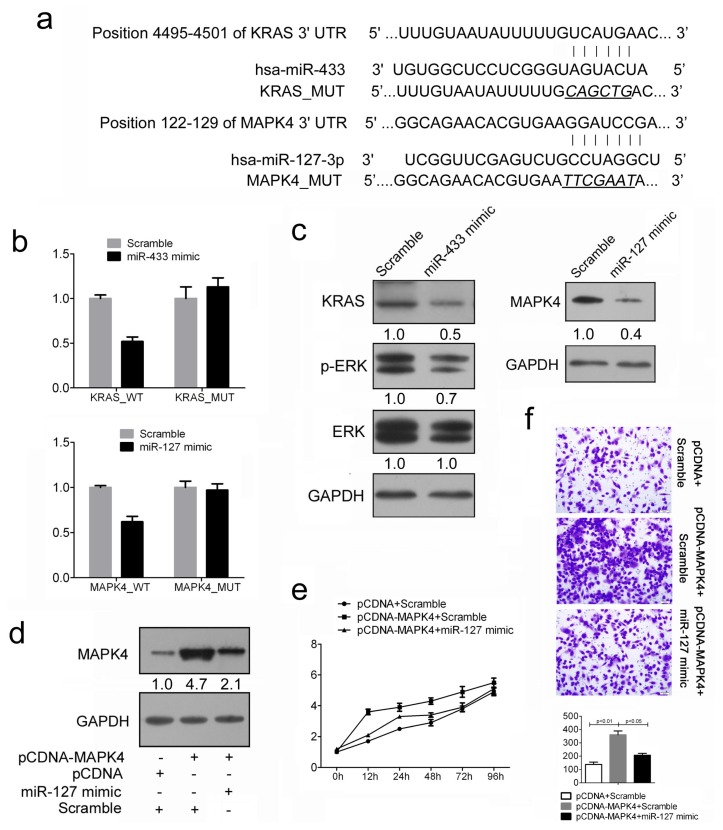
MiR-443 and miR-127 respectively target KRAS and MAPK4 in GC cells. (**a**) Schematic representation of KRAS and MAPK4 3′ UTRs showing putative miRNA target site; (**b**) Dual-luciferase analysis of reporter gene carrying KRAS and MAPK4 3′ UTR, co-transfected with miRNA mimic or Scramble; (**c**) Western blot analysis of KRAS and MAPK4 expression in HGC-27 cells transfected with miRNA mimic or Scramble. HGC-27 cells transfected with miR-433 mimics were also encountered with phosphorylated ERK (p-ERK) and total ERK analysis. Signals from Western blots are normalized to GAPDH, and the values, expressed as fractions with respect to the control, are indicated below; (**d**) Western blot analysis of MAPK4 in HGC-27 cells co-transfected with either miR-127 mimic (30 nM) or Scramble (30 nM) and 2.0 μg pCDNA-MAPK4 or pCDNA empty vector; (**e**) Cell proliferation assay of HGC-27 cells treated as described in d by using CCK-8; (**f**) Transwell analysis of HGC-27 cells treated as described in (d). The relative ratio of invasive cells per field was shown below.

To evaluate the possible regulation pathway of miR-433 in GC, we used two computational algorithms TargetScan and PicTar to predict the potential genes targeted by miR-433. Among them, KRAS was selected as an ideal candidate due to its well-characterized oncogenic roles in various cancers. The 3′UTR regions of KRAS was cloned into a luciferase reporter construct (Figure 4a). Reporter assays in 293T cells revealed miR-433-dependent repression of the KRAS 3′UTRs, and mutation of this site abrogated the reduction in luciferase activities (Figure 4b). In line with reporter assay, we observed a strong decrease of KRAS expression in presence of miR-433 mimics, whereas the scramble had no effects in HGC-27 cells (Figure 4c). Previous studies have shown the importance of the KRAS/ERK signaling pathway in the regulation of cell migration, invasion and metastasis [15–17]. To investigate whether miR-433 could affect GC cell phenotype through the KRAS/ERK pathway, we examined the phosphorylation level of ERK in HGC-27 cells overexpressing miR-1433. Cellular levels of p-ERK were significantly decreased in miR-433 mimics–transfected cells as compared with scramble-transfected cells (Figure 4c). However, no obvious difference was observed in total ERK level (Figure 4c). Considered together, these findings suggest that the accelerated gastric cancer cell growth and migration upon miR-433 overexpression were partially due to the attenuated ERK pathways.

### 2.5. MiR-127 Suppresses the Expression of MAPK4 in GC Cells

Similarly, MAPK4 was selected as the potential target of miR-127 for further assess (Figure 4a). As expected, both reporter assays and immunoblotting analysis showed the repression of miR-127 on MAPK4 3′UTRs, suggesting the direct inhibition of MAPK4 by miR-127 in GC cells (Figure 4b,c). As the oncogenic roles of MAPK4 in different cancers have been stated, thus, we reasoned that interfering with MAPK4 expression in GC cells might have inhibitory effect on cell growth. As expected, the ectopic expression of MAPK4 resulted in a marked increase in cell proliferation and cell migration (Figure 4d–f; see group pcDNA-MAPK4+Scramble and group pcDNA + Scramble). Next, we adapted a “rescue” strategy in order to examine the functional relevance of the miR-127/MAPK4 interaction in GC cells. The level of MAPK4 was reduced when miR-127 mimic were transfected after 24 h treatment of pcDNA-MAPK4 (Figure 4d). In agreement with the reduced expression of target proteins, decreased cell proliferation (Figure 4e), accompanied by decreased cell invasion (Figure 4f) were also observed in HGC-27 cells transfected with miR-127 mimic following treatment of pcDNA-MAPK4 construct. Thus, we conclude that the repression of cell growth by miR-127 is typically the consequence of decreased MAPK4 expression in GC.

### 2.6. Discussion

In the last few years, miRNAs have started a revolution in molecular biology and emerged as key players in various cellular processes [18–20]. For these reasons, it is extremely important to understand the physiological and disease-associated mechanisms underlying the regulation, function and mechanism of these small, single-stranded RNAs. In cancer, recent studies have shown that miRNA expression profiles are altered, giving rise to various malignancies [18]. Some miRNAs are down-regulated while others are over-expressed, suggesting that miRNAs can act as classic tumor suppressor genes or oncogenes, respectively. To date, several studies supporting this evidence have been published, for example, the first reports linking miRNAs and cancer showed that the deletion of a cluster of two miRNAs, miR-15a and miR-16-1, located on chromosome 13, was related to chronic lymphocytic leukemia (CLL) [21]; down-regulated let-7 expression in lung cancer was responsible for the tumor cell growth and survival [22]; miR-124a was specifically methylated in various cancer cells and served as a tumor suppressor [23]. In this study, we analyzed the expression of miR-443 and miR-127 in 86 GC patients and found that both of the two miRNAs were much lower in GC tissues compared with adjacent controls. The restored expression of miR-433 and miR-127 in HGC-27 and MGC-803 cells upon 5-Aza-CdR treatment indicated the hyper-DNA methylation on miR-433 and miR-127 locus in GC. Although miR-433 and miR-127 has been reported closely related to colorectal cancer, breast cancer, lung adenocarcinoma, esophageal squamous cell carcinoma, their function in gastric cancer remains to be determined.

In the present study, we showed that the restored expression of miR-433 and miR-127 in GC cell line HGC-27 resulted in reduced cell proliferation and inhibition of cell cycle progression. These results allow us to speculate that the silencing of miR-433 and miR-127 may provide a survival advantage to GC cells. On the other hand, metastasis is the major cause of morbidity and mortality from GC patients, therefore we subsequently detect the affects of miR-433 and miR-127 on cell migration and invasion. The introduction of miR-433 and miR-127 inhibited HGC-27 cell migration in wound healing assays, and suppressed cell invasion in transwell analysis, further confirming their association with the degree of GC malignance.

To further investigate the mechanism of miR-433 and miR-127 in GC, we predicted miRNA targets by various computer-aided algorithms. The genes that were predicted by all the three algorithm programs (TargetScan Release 4.0, RNA22 and miRanda) were picked out to be the candidate targets. In these genes, the v-Ki-ras2 Kirsten rat sarcoma viral oncogene homolog (KRAS) harboring miR-433 binding site and the mitogen-activated protein kinase 4 (MAPK4) harboring miR-127 binding site were regarded as potential target genes for miR-433 and miR-127 respectively. The overexpression of KRAS gene is an essential step in the development of many cancers including GC [24,25]. Here, we showed that miR-433 could repress the ERK pathway, whose activation was considered as a starting point in the development of GC, by direct inhibition of KRAS. MAPK4 mediates the signal of the growth factors, and thus regulates multiple cellular processes. The activation of MAPK4 signaling has been shown to be closely associated with the invasion ability of GC cells, and the p-MAPK4 expression level was significantly higher in diffuse type GCs, GCs with peritoneal metastasis, and GCs with lymph node metastasis [26,27]. Our results suggested that miR-127 could affect tumor migration and invasion through MAPK4 in highly differentiated GC. Since miR-127 and miR-433 are commonly regulated by nuclear receptor signaling [28,29], and our study showed that both miR-127 and miR-433 have similar function in GC cell growth and invasion, the clustered miR-127 and miR-433 may work in combination to reinforce their function. Hence, recognition that miRNAs arising from a same cluster can reinforce their action on similar cellular pathways may lead us to further investigate the mechanism associated with miR-433-127 cluster on the regulation of oncogenic cascades during GC tumorigenesis.

## 3. Experimental Section

### 3.1. Cell Cultures and Transfection

A total of 4 human gastric cancer cell lines MGC-803 (mucinous gastric carcinoma, poorly differentiated), HGC-27(metastatic lymph node, undifferentiated carcinoma), MKN-45(Signet ring carcinoma poorly differentiated), SGC-7901 (adenocarcinoma, moderately differentiated) were examined in this study. The MGC-803 cell line was purchased from the Cell Resource Center of Institute of Basic Medical Sciences, Chinese Academy of Medical Sciences and Peking Union Medical College (Beijing, China), and was propagated in Dulbecco’s modified Eagle medium (Gibco; Invitrogen; Life Technologies, Darmstadt, Germany), supplemented with 10% fetal bovine serum (FBS; PAA, Pasching, Austria) and streptomycin (100 μg/mL), penicillin (100 U/mL).The HGC-27, MKN-45, SGC-7901 cell lines were provided by American Type Culture Collection (ATCC, Manassas, VA, USA), and were maintained in RPMI 1640 medium (PAA) supplemented with 10% FBS (PAA). The human gastric cancer cell lines HGC-27 were transfected with miRNA mimics (GenePharma, Shanghai, China), negative control miRNA (Scramble; GenePharma; Shanghai, China) at a final concentration of 30 nmol/L using Dharmafect 1 (Dharmacon, Lafayette, CO, USA) in accordance with the manufacturer’s instructions.

### 3.2. Tissue Specimens

Gastric tumors and their morphologically normal tissue (located >3 cm away from the tumor) were obtained between November 2009 and November 2012 from 86 gastric cancer patients undergoing surgery at Cancer Hospital of Chinese Academy of Medical Sciences (CICAMS, *n* = 32) and Chinese PLA General Hospital (301 hospital, *n* = 34),The First Affiliated Hospital of Shanxi Medical University (*n* = 20). Tissue samples were cut into two parts, one was fixed with 10% formalin for histopathological diagnosis, and the other was immediately snap-frozen in liquid nitrogen, and stored at −196 °C in liquid nitrogen until RNA extraction. This group consisted of 72 males and 14 females with a median age of 59 years (range, 31–82 years). The use of the tissue samples for all experiments was approved by all the patients and by Ethics Committee of the institution. The characteristics of patients included are described in Table S1.

### 3.3. TaqMan RT-PCR for miRNA Expression

Total RNA was extracted from the cells and tissues with TRIzol reagent (Invitrogen, Carlsbad, CA, USA), MicroRNAs were quantitated by real-time PCR using TaqMan MicroRNA assay (Invitrogen, Carlsbad, CA, USA). First-strand complementary DNA (cDNA) synthesis was carried out from 1 μg of total RNA in 12 μL of final volume containing 2 M stem-loop primer, 10 mM dNTP Mix (Invitrogen, Carlsbad, CA, USA). The mix was plate at 65 °C for 5 min, and then mixed with 5× RT buffer, 0.1 M 0.1 M DTT, 200 U/μL MultiScribe reverse transcriptase and 40 U/μL RNase inhibitor (Invitrogen, Carlsbad, CA, USA). The mix was plate at 37 °C for 55 min, 70 °C for 15 min and then held at −20 °C. Real-time PCR was performed using a standard TaqMan PCR protocol. The 20 μL PCRs reactions included 1 μL of RT product, 1× Universal TaqMan Master Mix and 1× TaqMan probe/primer mix (Invitrogen, Carlsbad, CA, USA). The reactions were incubated in a 96-well plate at 95 °C for 30 s followed by 40 cycles of 95 °C for 3 s and 60 °C for 20 min. All RT reactions including no-template controls were run in triplicate. All mRNA quantification data were normalized to U6. The relative amount of transcript was calculated using the comparative Ct method.

### 3.4. 5-Aza-CdR and Trichostatin a Treatment of Cell Lines

Gastric cancer cell lines MGC-803 were treated with 5-aza-2′-deoxycytidine(5-Aza-CdR; Sigma-Aldrich, St. Louis, MO, USA) at 0.7 μmol/L, 1.5 μmol/L, 3 μmol/L; HGC-27 were treated with 5-Aza-CdR at 0.5 μmol/L, 1 μmol/L, 1.5 μmol/L for 3 days or 300 nmol/L trichostatin A (TSA; Sigma-Aldrich, St. Louis, MO, USA) for 24 hours, For the combination treatment, cells were first treated with 5-Aza-CdR for 48 h; then TSA (300 nmol/L) was added, and the cells were treated for an additional 24 h. Culture medium containing drug was replacement every 24 h. RNA of cell lines was purified with TRIzol reagent following the instruction from the manufacturer (Invitrogen, Carlsbad, CA, USA). cDNA synthesis was carried out as described earlier, and 1 mL of the diluted cDNA for each sample was amplified by RT-PCR using a protocol previously described.

### 3.5. Cell Proliferation and Cell Cycle Assay

Cells were incubated in 10% CCK-8 (DOJINDO, Kumamoto, Japan) diluted in normal culture medium at 37 °C until visual color conversion occurred. Proliferation rates were determined at 0, 24, 48, 72, 96 h after transfection. The absorbance of each well was measured with a microplate reader set at 450 nM and 630 nM. All experiments were performed in quadruplicate. Cell cycle analysis was performed on HGC-27 cell line 48 h after transfection with either miRNA mimics or Scramble.

Cells were harvested, washed twice with cold PBS, fixed in ice-cold 70% ethanol, and incubated with propidium iodide (PI) and RNase A, then analyzed by FACS. Each sample was run in triplicate.

### 3.6. Cell Migration and Invasion Assays

HGC-27 cells were grown to confluence on 12-well plastic dishes and treated with miRNA mimics or Scramble. Then 24 h after transfection, linear scratch wounds (in triplicate) were created on the confluent cell monolayers using a 200 μL pipette tip. To remove cells from the cell cycle prior to wounding, cells were maintained in serum-free medium. To visualize migrated cells and wound healing, images were taken at 0, 24, 48 h. A total of ten areas were selected randomly fromeach well and the cells in three wells of each group were quantified.

For the invasion assays, after 24 h transfection, 1 × 105 cells in serum-free media were seeded onto the transwell migration chambers (8 μm pore size; Millipore, Zürich, Switzerland) which coated with the upper chamber of an insert coated with Matrigel (Sigma-Aldrich, St. Louis, MO, USA). Media containing 20% FBS were added to the lower chamber. After 24 h, the noninvading cells were removed with cotton wool, Invasive cells located on the lower surface of the chamber were stained with May-Grunwald-Giemsa stain (Sigma-Aldrich, St. Louis, MO, USA) and counted using a microscope (Olympus, Tokyo, Japan). Experiments were independently repeated three times.

### 3.7. Protein Isolation and Western Blotting

Cells were washed twice in ice-cold phosphate-buffered saline, and lysed in JS buffer (50 mM HEPES pH 7.5 containing 150 mM NaCl, 1% Glycerol, 1% Triton X-100, 1.5 mM MgCl_2_, 5 mM EGTA, 1 mM Na_3_VO_4_ and protease inhibitor cocktail). Protein concentration was determined by the BCA Protein Assay Kit (Bio-Rad, Milan, Italy) and equal amounts of proteins were analyzed by SDS–PAGE (10% acrylamide). Gels were electroblotted onto nitrocellulose membranes (Millipore, Bedford, MA, USA). For immunoblot experiments, membranes were blocked for 2 h with 5% non-fat dry milk in Tris-buffered saline containing 0.1% Tween-20, and incubated at 4 °C over night with primary antibody. Detection was performed by peroxidase-conjugated secondary antibodies using the enhanced chemiluminescence system. Primary antibodies used were: GAPDH from Zhong- Shan JinQiao (Beijing, China); ERK and phospho-ERK (p-ERK); KRAS and MAPK4 were from New England Biolabs.

### 3.8. Rescue Assays of MAPK4 Gene Expression

The full length MAPK ORF were PCR amplified and cloned into pCDNA3.1 to generate the pcCDNA- MAPK4 constructs, which were used in the rescue assays. Sequences of primers used for full length amplification are shown in Table S2. HGC-27 cells in 6-well plates were first transfected with miR-127 mimic or scramble (30 nM). After 24 h in culture, these H cells were then co-transfected with either miR-127 mimic (30 nM) and 2.0 μg pCDNA-MAPK4 or pCDNA empty vector. Cells were harvested at indicated times and assayed as required.

### 3.9. Statistics

Student’s *t*-test (two-tailed) was performed to analyze the data. *p*-values < 0.05 were considered significant.

## 4. Conclusions

In conclusion, expression and functional studies suggest that both miR-433 and miR-127 have tumor suppressive function in GC via targeting MAPK4 and KRAS respectively. Our experiments also documented the lower expression of miR-433 and miR-127 was associated with higher grade and later stage tumors. Reduction of cell proliferation, cell cycle progression, cell migration and invasion by miRNA mimics indicates that such strategy may serve as a basis for the development of new potential therapies for GC patients.
